# High-Protein Diet Prevents Glucocorticoid-Induced Fat Mass Accumulation and Hyperglycemia

**DOI:** 10.3390/ijms26094212

**Published:** 2025-04-29

**Authors:** Susan J. Burke, Heidi M. Batdorf, Maggie P. Ducote, Thomas M. Martin, Michael D. Karlstad, Robert C. Noland, Sujoy Ghosh, Christopher D. Morrison, J. Jason Collier

**Affiliations:** 1 Laboratory of Immunogenetics, Pennington Biomedical Research Center, Baton Rouge, LA 70808, USA; 2 Department of Biological Sciences, Louisiana State University, Baton Rouge, LA 70803, USA; 3 Laboratory of Islet Biology and Inflammation, Pennington Biomedical Research Center, Baton Rouge, LA 70808, USA; thomas.martin@pbrc.edu; 4 Department of Surgery, Graduate School of Medicine, University of Tennessee Health Science Center, Knoxville, TN 37920, USA; 5 Skeletal Muscle Metabolism Laboratory, Pennington Biomedical Research Center, Baton Rouge, LA 70808, USA; 6 Laboratory of Computational Biology, Pennington Biomedical Research Center, Baton Rouge, LA 70808, USA; 7 Laboratory of Neurosignaling, Pennington Biomedical Research Center, Baton Rouge, LA 70808, USA

**Keywords:** diabetes, glucocorticoids, protein, transcriptomics

## Abstract

Glucocorticoid-induced diabetes is the most common form of drug-induced hyperglycemia. In addition, chronic exposure to glucocorticoids promotes lean mass loss and fat mass accumulation. In this study, we hypothesized that a high-protein diet (60% kcal; HPD) would help to offset sarcopenia during oral administration of corticosterone to C57BL/6J mice. Carbohydrates were reduced in the HPD to ensure it was isocaloric with the normal-protein diet (20% kcal; NPD). We found that the HPD prevented fat mass accumulation but did not protect against reductions in lean mass in both male and female mice. Mice consuming a HPD did not develop hyperglycemia, while mice given the NPD developed hyperglycemia within two weeks. The HPD diet did not improve insulin sensitivity in response to glucocorticoids but did alter gene expression patterns in adipose tissue and liver measured by RNA sequencing. We conclude that a HPD diet may be beneficial to limit rises in blood glucose and adipose tissue accrual during glucocorticoid therapy.

## 1. Introduction

Glucocorticoids (GCs) are endogenously produced hormones that regulate a variety of biological processes, including inflammation, metabolism, bone dynamics, and cardiovascular outcomes. These steroid hormones act through the glucocorticoid receptor (GR), an intracellular receptor belonging to the nuclear hormone receptor superfamily [1,2]. In addition to the physiological actions of endogenous GCs, synthetic glucocorticoids are used clinically to treat numerous diseases, including asthma, chronic obstructive pulmonary disease, cancers, rare disorders with inflammatory and autoimmune origins, and more common pathologies associated with autoimmunity [3]. In addition, most, if not all, tissue transplant recipients require long-term glucocorticoid therapy to prevent rejection of the grafted tissue.

The continued use of GCs is associated with an increased risk of diabetes, a condition often referred to as “steroid-induced diabetes” [4,5,6]. This is one of the most common forms of drug-induced diabetes, with chronic use of GCs contributing to millions of cases of new-onset diabetes annually [7]. The risk of hyperglycemia rises with the duration and dose of GC interventions [8,9]. For example, 17% and 60% of patients with kidney and liver transplants, respectively, became diabetic while using a glucocorticoid to prevent organ rejection [10]. Moreover, nearly 13% of lupus patients became diabetic during glucocorticoid therapy [11]. While the many side effects of GCs are well known [12], it is not currently understood how to best mitigate the adverse effects in situations where chronic GC therapy is recommended or absolutely required for therapeutic benefit.

Some of the side effects of GCs include insulin resistance, skeletal muscle atrophy, and fat mass gain in addition to the risk of hyperglycemia. In this study, we investigated the hypothesis that a high-protein diet could protect against these specific adverse outcomes associated with prolonged exposure to GCs. The rationale was based on evidence from human studies that a high-protein diet promoted lean mass, restricted fat mass, and protected against pathologies evident in humans with existing diabetes [13,14,15]. Accordingly, we used a well-established pre-clinical model of chronic glucocorticoid exposure [16,17,18] to test whether a high-protein diet (HPD) could provide therapeutic benefit against known side effects of GC exposure.

We made several novel observations: (1) HPD prevented progression to hyperglycemia in mice receiving oral corticosterone (cort) administration. (2) HPD did not protect against losses in lean mass but did restrict the cort-induced accumulation of fat mass. (3) HPD did not prevent the onset of insulin resistance. (4) HPD altered the expression of metabolic-pathway-associated genes in adipose tissue and liver during cort exposure, which is correlated with protection against hyperglycemia.

## 2. Results

### 2.1. High-Protein Diet Protects Against Glucocorticoid-Induced Hyperglycemia in Male Mice

**Figure 1:**
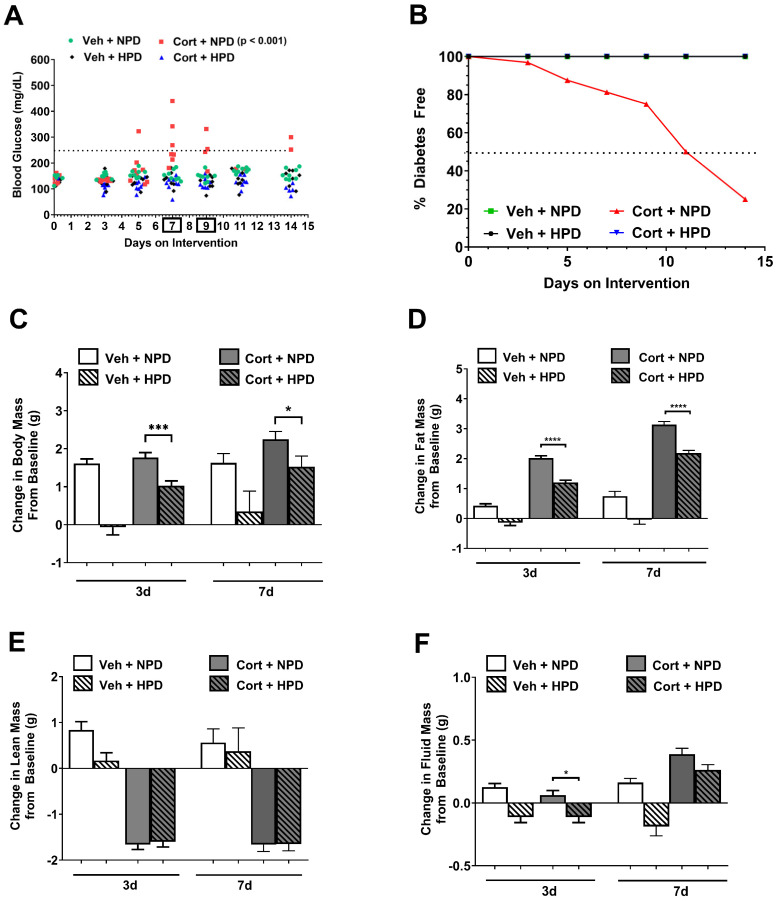
(**A**) Blood glucose values taken over a 14-day period in 10-week-old male mice given 100 μg/mL corticosterone (Cort) or 1% ethanol vehicle (Veh) control in the drinking water while being administered either a normal-protein diet (NPD) or a high-protein diet (HPD). The two-way ANOVA *p*-value for Cort NPD relative to the other groups for days 7 and 9 (boxed on *x*-axis) is shown. (**B**) Values in panel (**A**) were used to determine the % of animals that remained diabetes free over this 14 day period. (**C**–**F**) Body composition was assessed in an identically treated but separate cohort at 0, 3, and 7 days. The change in body mass (**C**), fat mass (**D**), lean mass (**E**), and fluid mass (**F**) was calculated for 3 and 7 days of intervention. * *p* ≤ 0.05, *** *p* ≤ 0.001, **** *p* ≤ 0.0001. (**A**,**B**) n = 21–23/group for blood glucose values (assessed in multiple cohorts of mice at both PBRC and UT). (**C**–**F**) n = 8/group.

Chronic exposure to glucocorticoids leads to insulin resistance and the development of hyperglycemia [17,18,19,20,21,22]. We tested the hypothesis that a diet with high protein and reduced carbohydrates could be protective in the context of glucocorticoid exposure. We found that mice consuming a high-protein diet (HPD; 60% kcal) did not develop hyperglycemia when compared with mice fed a diet matched in fat content but with normal protein (20% kcal; Figure 1A,B). The dotted line in Figure 1A represents the threshold for hyperglycemia in our studies (two consecutive daily measurements of ≥250 mg/dL. Note in Figure 1B that all groups except Cort + NPD overlay. The dotted line in Figure 1B represents 50% diabetes free. The HPD had less carbohydrates than the NPD to ensure the two diets matched in calories. We hypothesized that the HPD would protect against losses in lean mass that are normally observed during chronic glucocorticoid exposure. We found that while total body mass was not significantly different over seven days in response to cort in mice eating the NPD, mice eating the HPD showed reduced body mass on both vehicle and cort when compared with mice on the NPD (Figure 1C). In addition, the HPD reduced fat mass gain (Figure 1D). Our original hypothesis that lean mass loss that normally occurs during cort exposure would be protected by HPD intervention was not supported (Figure 1E). There were minimal changes in fluid mass with a slight but significant reduction between the NPD and the HPD in mice given cort (Figure 1F).

### 2.2. High-Protein Diet Enhances Circulating Glucagon and Suppresses Acute Upregulation of FGF21 During Corticosterone Exposure in Male Mice

**Figure 2:**
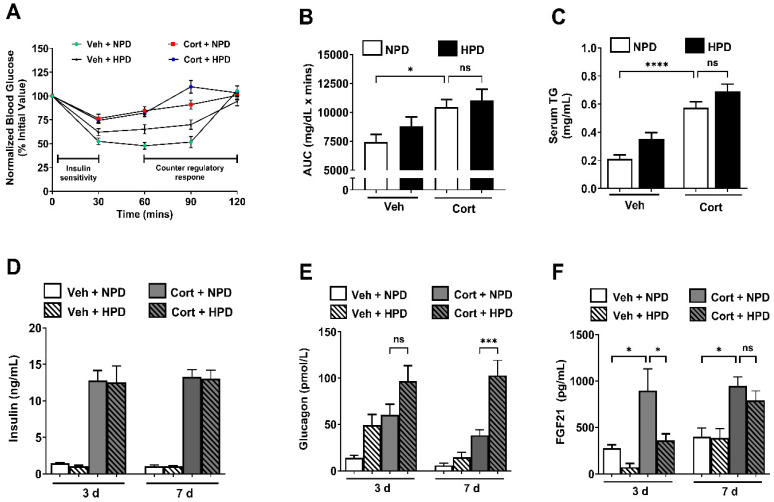
(**A**) Insulin tolerance test (ITT) in 10-week-old male mice given 100 μg/mL corticosterone (Cort) or 1% ethanol vehicle (Veh) control in the drinking water while being administered either a normal-protein diet (NPD) or a high-protein diet (HPD) for 7 days. ITT is represented as normalized blood glucose where the baseline value is set to 100% for all samples. (**B**) Area under the curve (AUC) for ITT shown in panel (**A**). (**C**–**F**) Serum factors including triglycerides (**C**), insulin (**D**), glucagon (**E**), and FGF21 (F) were assessed at 3 (**D**–**F**) and/or 7 (**C**–**F**) days post-intervention. ns = not significant, * *p* ≤ 0.05, *** *p* ≤ 0.001, **** *p* ≤ 0.0001. (**A**,**B**) n = 21–23/per group for ITT in (**A**) (assessed in multiple cohorts of mice at both PBRC and UT). (**C**–**F**) n = 8/group.

The onset of hyperglycemia is typically preceded by insulin resistance in response to glucocorticoid administration in rodents and humans [23,24,25,26]. Therefore, we next performed insulin tolerance tests to assess whole-body insulin sensitivity in the context of the NPD and the HPD. We found that cort-treated mice fed a HPD did not significantly improve insulin sensitivity (Figure 2A,B) despite improving glycemia (Figure 1A,B). The HPD also did not significantly alter serum TGs (Figure 2C) or insulin (Figure 2D). However, circulating glucagon levels were much greater in mice consuming the HPD relative to those given the NPD (Figure 2E). FGF21, another hormone related to protein intake [27,28], was induced in response to cort treatment and suppressed by the HPD early (3 days on diet). FGF21 in circulation was back to near normal levels by day 7 on the diets (Figure 2F).

### 2.3. High-Protein Diet Prevents Fat Mass Accumulation in Female Mice

**Figure 3:**
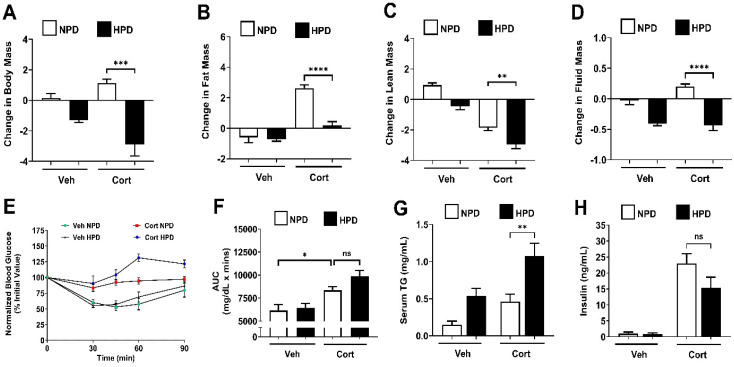
(**A**–**D**) Body composition was assessed in 10-week-old female mice given 100 μg/mL corticosterone (Cort) or 1% ethanol vehicle (Veh) control in the drinking water while being administered either a normal-protein diet (NPD) or a high-protein diet (HPD) for 7 days. The change in body mass (**A**), fat mass (**B**), lean mass (**C**), and fluid mass (**D**) over the 7-day intervention period. Following the 7-day intervention, insulin tolerance was assessed by ITT (**E**) with respective AUC in (**F**), and serum measurements of TGs (**G**) and insulin (**H**) were evaluated. ns = not significant, * *p* ≤ 0.05, ** *p* ≤ 0.01, *** *p* ≤ 0.001, **** *p* ≤ 0.0001. n = 8/group.

Glucocorticoids promote fat mass accumulation in rodents and humans [17,18,22,29]. In female mice, a HPD reduced overall body fat over time and prevented the cort-induced accrual of whole-body fat mass (Figure 3A,B). As we observed in male mice, a HPD did not fully protect female mice against losses in lean mass (Figure 3C). Fluid mass was also lower in female mice consuming the HPD (Figure 3D). Similar to male mice, female mice showed reduced insulin sensitivity in response to cort regardless of diet (Figure 3E,F). Serum TGs were elevated in response to cort on the HPD compared to the NPD (Figure 3G) while circulating insulin was elevated by cort regardless of diet (Figure 3H).

### 2.4. Whole-Body Fuel Usage in Male and Female Mice Is Altered in Response to Diet

**Figure 4:**
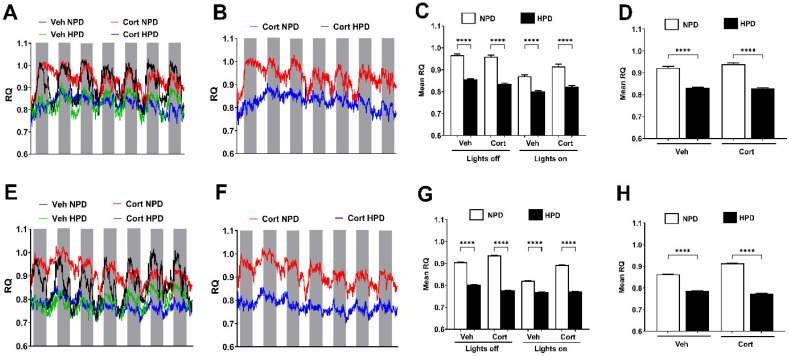
(**A**–**H**) Respiratory quotient (RQ) was evaluated in 10-week-old male (**A**–**D**) and female (**E**–**H**) mice receiving 100 μg/mL corticosterone (Cort) or 1% ethanol vehicle (Veh) control in the drinking water while being administered either a normal-protein diet (NPD) or a high-protein diet (HPD) for 7 days. (**A**,**E**) RQ values during both light on (white bars) and light off (gray bars) cycles over a 7-day period for all groups. (**B**,**F**) RQ values during both light on and off cycles over a 7-day period for Cort groups only. (**C**,**G**) Mean RQ calculated for both light on and off cycles for a 7-day period. (**D**,**H**) Mean RQ for a 7-day period. **** *p* ≤ 0.0001. n = 8/group.

To maintain isocaloric conditions between the diets, the HPD has less carbohydrates than the NPD to account for the increase in protein as an energy source. We observed that reductions in carbohydrates in the HPD were reflected by changes in the respiratory quotient (RQ), as shown in Figure 4. This phenotype of reduced carbohydrate usage was maintained whether the RQ was measured in the light versus the dark cycle (Figure 4) and in both male (Figure 4A–D) and female mice (Figure 4E–H).

### 2.5. Energy Expenditure (EE) in Male and Female Mice Is Driven by Changes in Protein Content Within the Diet

**Figure 5:**
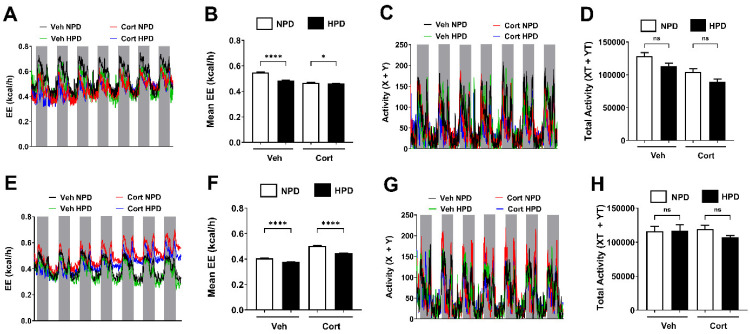
(**A**,**B**,**E**,**F**) Energy expenditure (EE) was determined in 10-week-old male (**A**,**B**) and female (**E**,**F**) mice receiving 100 μg/mL corticosterone (Cort) or 1% ethanol vehicle (Veh) control in the drinking water while being administered either a normal-protein diet (NPD) or a high-protein diet (HPD) for 7 days. (**A**,**E**) EE values during both light on (white bars) and light off (gray bars) cycles over a 7-day period for all groups. (**B**,**F**) Mean EE calculated for both light on and off cycles for a 7-day period. (**C**,**G**) Activity (X and Y beam breaks) of male (**C**) and female (**G**) mice during both light on (white bars) and light off (gray bars) cycles over a 7-day period for all groups. (**D**,**H**) Total activity for male (**D**) and female (**H**) mice across all light cycles during the 7-day intervention. ns = not significant, * *p* ≤ 0.05, **** *p* ≤ 0.0001. n = 8/group.

We next measured energy expenditure over a seven-day period in mice consuming either the NPD or the HPD in the presence and absence of cort. We found that the HPD significantly reduced the total EE (Figure 5). This was true in both male and female mice. By contrast, total spontaneous physical activity was not significantly different between the diets or the mice exposed to cort during this one-week period (Figure 5).

### 2.6. RNA-Seq Reveals Distinct Changes in Transcript Abundance in eWAT and Liver

**Figure 6:**
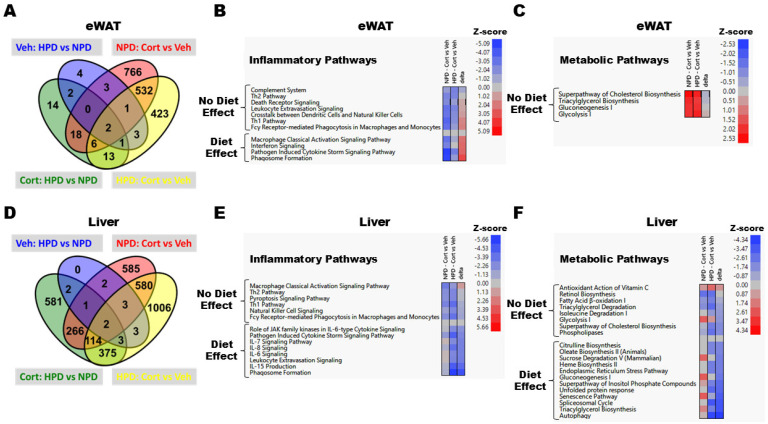
RNA-seq analysis was performed in epididymal white adipose tissue (eWAT) and liver from mice given 100 μg/mL corticosterone (Cort) or 1% ethanol vehicle (Veh) control in the drinking water. Venn diagrams and heatmaps show the number of genes that were significantly regulated in each group comparison, as well as genes that overlapped amongst group comparisons, in eWAT (**A**) and liver (**D**). RNA-seq data were used to identify inflammatory and metabolic pathways that were regulated by Cort in eWAT ((**B**) and (**C**), respectively) and liver ((**E**) and (**F**), respectively) of mice fed a normal-protein diet (NPD—Cort vs. Veh) or a high-protein diet (HPD—Cort vs. Veh). Heatmaps show the predicted activation status (i.e., Z-score) of the listed pathway. Diet effects were estimated as the difference of the Z-scores between the HPD: Cort vs. Veh minus NPD: Cort vs. Veh groups, which is represented as “delta” on the heatmaps. n = 3–4/group.

The HPD had a limited impact on genomic changes in epididymal white adipose tissue (eWAT) regardless of whether mice received cort (56 genes altered; green) or veh control (16 genes altered; blue; Figure 6A). By contrast, cort promoted substantial genomic changes in eWAT regardless of whether mice were fed the NPD (1328 genes altered; red) or the HPD (981 genes altered; yellow). As expected, GR agonism by cort significantly downregulated inflammatory pathways in eWAT; however, the HPD blunted the response of a subset of these pathways (macrophage activation, interferon signaling, pathogen-induced cytokine storm signaling, and phagosome formation), which is labeled as “Diet Effect” on the heatmaps and represents a difference of greater than two in the delta of the Z-scores between group comparisons (Figure 6B). In line with the known effects of glucocorticoids on adipose tissue, there were significantly upregulated pathways involved in cholesterol biosynthesis, triacylglycerol synthesis, gluconeogenesis, and glycolysis in eWAT (Figure 6C). However, the protein composition of the diet did not influence the regulation of these metabolic pathways in response to cort exposure (Figure 6C).

Similar to findings in eWAT, the HPD did not significantly change the transcriptome in the liver of veh-treated mice (16 genes altered, blue; Figure 6D). However, in stark contrast to eWAT, feeding mice a HPD did substantially impact the hepatic transcriptomic response in cort-treated mice (1344 genes altered; green). Cort significantly altered hepatic gene expression in mice fed the NPD (1583 genes altered; red), but the response was even more robust in mice fed a HPD (2086 genes altered; yellow). Upon further analysis, cort inhibited several inflammatory pathways in the liver, and the HPD appeared to further potentiate many of the anti-inflammatory effects of cort in the liver beyond those that were observed in mice fed the NPD (Figure 6E—“Diet Effect”).

Cort also impacted the activation status of several metabolic pathways in the liver, with dietary protein composition contributing to a subset of such pathways (Figure 6F). Dietary protein did not significantly impact the cort-induced regulation of fatty acid oxidation, triglyceride degradation, glycolytic, or cholesterol biosynthesis pathways (Figure 6F—“No Diet Effect”). However, the HPD significantly reduced the predicated activation status of several key metabolic pathways, including autophagy, senescence, unfolded protein response, ER stress, triglyceride/oleate synthesis, gluconeogenesis, and sucrose degradation (Figure 6F—“Diet Effect”).

## 3. Discussion

Glucocorticoids (GCs) are ligands for the intracellular glucocorticoid receptor. Activation of the GR by GCs is a very effective anti-inflammatory strategy that has important utility in controlling a wide variety of human diseases. However, the chronic use of GCs is associated with severe metabolic side effects, including insulin resistance and a markedly enhanced risk for diabetes [12,30]. Because GCs are administered systemically, and most, if not all, tissues express the GR, there are a multitude of outcomes associated with GR agonism. Herein, we report key metabolic outcomes using a preclinical model of glucocorticoid exposure, which includes the onset of hyperglycemia, and how manipulating the dietary protein content influences whole-body and tissue-level responses in vivo.

Our initial hypothesis that increases in dietary protein content would protect against the known sarcopenic actions of glucocorticoids was not supported (Figure 1E). However, consumption of the HPD did suppress fat mass accrual in response to glucocorticoid exposure (Figure 1D). We used whey protein in the dietary formulation because it is more rapidly absorbed and has a higher fractional synthetic rate compared with casein [31]. A separate study reported increases in lean mass and reduced fat mass in mice consuming a casein-based high-protein diet [32]. Our data agree and extend these findings to the impact of the HPD on mice exposed to corticosterone.

Regarding changes in fat mass, we note that the HPD suppressed fat mass accumulation in both the vehicle-treated group and in mice exposed to cort (Figure 1D). Our data in the vehicle-treated mice are in line with previous studies [32]. The reduction in fat mass accrual appears to occur in the high-protein groups regardless of whether the protein was provided as casein [32] or whey (present study). In efforts to keep the diets isocaloric, the HPD has reduced carbohydrates as an energy source (see Supplementary Table S1). Accordingly, the reduced body fat and lowering of glycemia observed in this study could reflect reduced sugar in the diet, increased protein, or both. Regardless, our data are consistent with similar diets improving HbA1C and glycemic control in human studies [33,34,35]. Moreover, we note that the converse is also true: consumption of excess sucrose promotes fat mass accumulation and increased risk of cardiovascular disease [36,37,38]. We interpret these data collectively to indicate that multiple mechanisms exist to explain the beneficial effects of consuming high protein coupled with reduced carbohydrates. At present, the effects of these two dietary variables, as well as long-term outcomes associated with this type of diet, cannot be determined by the present study outcomes.

In terms of the mechanism of the dietary effects to prevent hyperglycemia, the liver of mice consuming the HPD showed reduced expression of genes related to both sucrose degradation and gluconeogenesis (Figure 6F), which could reflect less glucose production by the liver in response to cort. The decrease in dietary carbohydrates is also reflected in the respiratory quotient (RQ) in both male and female mice (Figure 4). Importantly, the difference in dietary composition associated with high protein could thus be highly relevant to protection against hyperglycemia. Our findings are consistent with recommendations by the American Diabetes Association to reduce carbohydrates in the diet as a strategy to maintain glycemic control. In line with this recommendation, studies using low-carbohydrate diets have shown promise in diabetes [39]. In addition, high-protein diets provide greater satiety [40], thus offering a potential opportunity to control overconsumption of calories and thus glycemia by simultaneously reducing carbohydrates while increasing protein intake. Finally, the increase in circulating glucagon in response to high-protein feeding (Figure 2E) may lower glycemia through: (1) effects in the brain [41] and (2) by increasing both lipolysis and glucose uptake into adipose tissue [42]. The reported effects of glucagon help to explain the lowering of glycemia and reduction in fat mass observed with high-protein dietary intervention.

Using an RNA-seq-based approach to examine global transcript changes in an unbiased manner, we observed contrasting effects of the HPD in eWAT relative to the liver (Figure 6). While the HPD enhanced the effect of cort to suppress genes linked with inflammation in the liver, it had the opposite effect in eWAT. This tissue-specific difference may be due to inflammation being required for healthy adipose mass expansion [43]. Thus, a heightened gene expression response in adipose tissue could reflect a compensatory effect driven by the reductions in whole-body fat mass (Figure 1D). At present, we do not know whether these observations are due to the increased protein content, reduced carbohydrate content, or both. It is also possible that the different macronutrients contribute discrete outcomes to each tissue in terms of supporting or opposing GR transcriptional activity. Consequently, it remains to be determined whether nutrient-sensing mechanisms overlap with or directly regulate glucocorticoid receptor activity; however, that possibility seems likely. In addition, the change in circulating hormonal factors, such as insulin, glucagon, and FGF21, may also regulate glucocorticoid receptor activity and overall responsiveness to agonist ligands. These possibilities remain an area of active investigation to understand both ligand-dependent and dietary-mediated responses of glucocorticoids.

In summary, we have explored the outcomes associated with HPD intervention in a preclinical model of glucocorticoid exposure. We found that the incidence of hyperglycemia and whole-body fat mass was reduced, while there was no protection against cort-driven lean mass loss or insulin resistance. The reduction in cort-driven hyperglycemia in response to the HPD was supported by a decline in the expression of genes encoding enzymes of the gluconeogenesis pathway in the liver. Overall, lowering carbohydrates and increasing protein intake may be a viable strategy to control blood glucose levels for those requiring continuous glucocorticoid regimens to regulate chronic disease symptoms.

## 4. Materials and Methods

### 4.1. Experimental Mice and Reagents

All procedures were approved by the Institutional Animal Care and Use Committees at the Pennington Biomedical Research Center and the University of Tennessee, Knoxville. Male and female C57BL/6J mice (stock no. 000664) were obtained from the Jackson Laboratories (Bar Harbor, ME, USA) at 7 weeks of age. Mice were group housed and allowed at least 7 days to adjust to the light/dark cycle (12 h light/12 h dark) and room temperature (22 °C ± 1 °C). During this acclimation period, they had unrestricted access to water and Lab Diet 5015 (Lab Supply, Ft. Worth, TX; catalog no. 0001328).

At 8 weeks of age, mice were switched from Rodent Diet 5015 (26% fat kcal; LabDiet) to a purified diet containing 20% kcal from whey protein isolate, 70% kcal from carbohydrates (sucrose matched with high-protein diet below), and 10% kcal from fat (NPD; Research Diets, New Brunswick, NJ). Dietary formulations are provided in Supplementary Table S1. Mice were also switched from standard hydropacs to water bottles to acclimate drinking via water bottles for the study. Mice were randomized into four study groups using baseline assessments of blood glucose and body weight to confirm no significant metabolic differences existed among groups prior to starting the intervention. Experiments were initiated when mice reached 10 weeks of age. On study day zero, animals were either maintained on NPD or switched to an isocaloric rodent diet containing 60% kcal from whey protein isolate, 30% kcal from carbohydrates (reduced corn starch to be isocaloric with NPD), and 10% kcal from fat (HPD; Research Diets). Likewise, on day zero, mice were given either vehicle or corticosterone (cort; catalog no. 27840; MilliporeSigma, St. Louis, MO, USA), as previously described [17,18,22]. Briefly, cort was dissolved in 100% ethanol and diluted with water to create a 1% ethanol solution, resulting in a final cort concentration of 100 μg/mL in the drinking water. Mice in the vehicle control group received 1% ethanol in their drinking water. Both vehicle and corticosterone solutions were changed twice a week. Upon completion of studies, animals were fasted for 4 h, anesthetized by CO_2_ asphyxiation, and then euthanized by decapitation. Fat depots and liver tissue were snap-frozen in liquid nitrogen. Trunk blood was harvested, and the serum fraction was collected. Multiple cohorts of mice were required to complete the studies described herein. 

### 4.2. Blood Glucose, Body Composition, and Metabolic Cage Analysis

Non-fasting blood glucose measurements were obtained from the tail vein using the Bayer Contour Glucometer (Bayer, Whippany, NJ, USA). Measurements of body mass and composition (fat, lean, and fluid mass) were assessed by NMR using a Minispec LF110 Time-Domain NMR system (Bruker, Billerica, MA, USA). Mice were classified as diabetic if blood glucose exceeded 250 mg/dL on two consecutive days, whereupon they were necropsied. For measurements of respiratory quotient (RQ), energy expenditure (EE), and spontaneous locomotor activity, a cohort of male mice was monitored for a 7-day period using Promethion metabolic cages (Sable Systems, North Las Vegas, NV, USA). Acclimation using training cages followed by metabolic cage analyses has been described [38].

### 4.3. Insulin Tolerance Test (ITT) and Serum ELISAs

Insulin sensitivity was assessed by ITT following a 2 h fast. Mice were given an intraperitoneal injection of 1 U/kg lean mass of insulin (Humulin R; Eli Lilly & Co., Indianapolis, IN, USA). Blood glucose measurements were taken at 0, 30, 45, 60, 90, and 120 min. Serum triglycerides were calculated using the Serum Triglyceride Determination Kit from MilliporeSigma. Serum insulin (Cat # 10-1247-10) and glucagon (Cat # 10-1281-01) were measured using ELISA kits from Mercodia (Uppsala, Sweden). Mouse FGF21 was quantified using an ELISA kit from R&D Systems (Cat # MF2100; Minneapolis, MN, USA).

### 4.4. RNA Isolation, cDNA Synthesis, and RNA-Seq

Total RNA was isolated from 30 mg of tissue using an RNeasy Mini Kit (Qiagen, Hilden, Germany). Total RNA was reverse transcribed into cDNA using the iScript cDNA Synthesis Kit (Bio-Rad, Hercules, CA, USA). RNA content and quality (260/280 ratio range 1.9–2.1) were assessed using a NanoDrop 1000 (Thermo Fisher Scientific, Waltham, MA, USA). RNA Integrity Number (RIN) was determined using the 2100 Bioanalyzer (Agilent Technologies, Santa Clara, CA, USA) and found to be between 8 and 10 for all RNA samples. Total RNA was assessed using the Bioanalyzer RNA 6000 Assay (Agilent) to verify high-quality RNA. RNA-Seq Library Construction was performed using the Lexogen QuantSeq 3′ mRNA-Seq V2 Library Prep Kit FWD with Unique Dual Indices (Cat No: 191.96; Vienna, Austria). Libraries were confirmed using BioAnalyzer High Sensitivity DNA Assay (Agilent). The ~275 bp libraries were pooled in equimolar amounts and sequenced on the Illumina NextSeq2000 (San Diego, CA, USA). All procedures for analyses have been described previously [44,45].

The SAGE dataset has been uploaded to the Gene Expression Omnibus website (https://www.ncbi.nlm.nih.gov/geo/, uploaded on 30 January 2025; accession number GSE288428). The PCA analysis is located in Supplementary Figure S1. After initial analysis, gene counts were log_2_ transformed and those with a pAdj < 0.05 between groups of interest were used for downstream analyses. A curated list of significantly regulated genes containing the gene name, pAdj value, and log_2_ fold change (logFC) value for each comparison were input into Ingenuity Pathway Analysis software (Qiagen, www.qiagen.com/ingenuity, accessed on 7 March 2025) to identify changes in canonical pathways. Canonical pathways with a −log(*p*-value) > 2 were considered to be significantly regulated, and the Z-score (i.e., predicted activation status) was used to generate heatmaps using JMP 17 software.

Heatmaps show the effects of corticosterone vs. vehicle (i.e., cort effects) in mice fed the normal-protein diet (NPD) versus the high-protein diet (HPD). Diet effects were estimated by comparing the Z-score values of regulated canonical pathways between each of these groups (represented as “delta” on the heatmaps), and differences that were either <−2 or >+2 were considered to be a dietary effect.

### 4.5. Statistics

Statistical analysis was performed using GraphPad Prism 10.1.1 (GraphPad Software, La Jolla, CA, USA). All data were analyzed by two-tailed Student’s *t*-test, one-way or two-way analysis of variance (ANOVA) with Tukey’s post-hoc test. Datasets were tested for outliers using the Rout method (Q = 1%). Data are reported as means ± SEM.

## Data Availability

The SAGE dataset has been uploaded to the Gene Expression Omnibus website (https://www.ncbi.nlm.nih.gov/geo/) using accession number GSE288428. The PCA analysis is located in Supplementary Figure S1.

## Supplementary Materials

The following Supporting Information can be downloaded at: https://www.mdpi.com/article/10.3390/ijms26094212/s1.

## Abbreviations

The following abbreviations are used in this manuscript:

HPD	High-Protein Diet
NPD	Normal-Protein Diet
GC	Glucocorticoid
Cort	Corticosterone
FGF21	Fibroblast Growth Factor 21
Veh	Vehicle
AUC	Area Under the Curve
ITT	Insulin Tolerance Test
RQ	Respiratory Quotient
EE	Energy Expenditure
eWAT	epididymal White Adipose Tissue
GR	Glucocorticoid Receptor
NMR	Nuclear Magnetic Resonance
