# Supplementary material for: High-Protein Diet Prevents Glucocorticoid-Induced Fat Mass Accumulation and Hyperglycemia
Source: Int J Mol Sci. 2025 Apr 29;26(9):4212. doi: 10.3390/ijms26094212 (PMC12071877; doi:10.3390/ijms26094212)
Supplement: Supplementary file 1 [file ijms-26-04212-s001.zip › ijms-3559219-supplementary.pdf]

SUPPLEMENTAL FIGURE S1

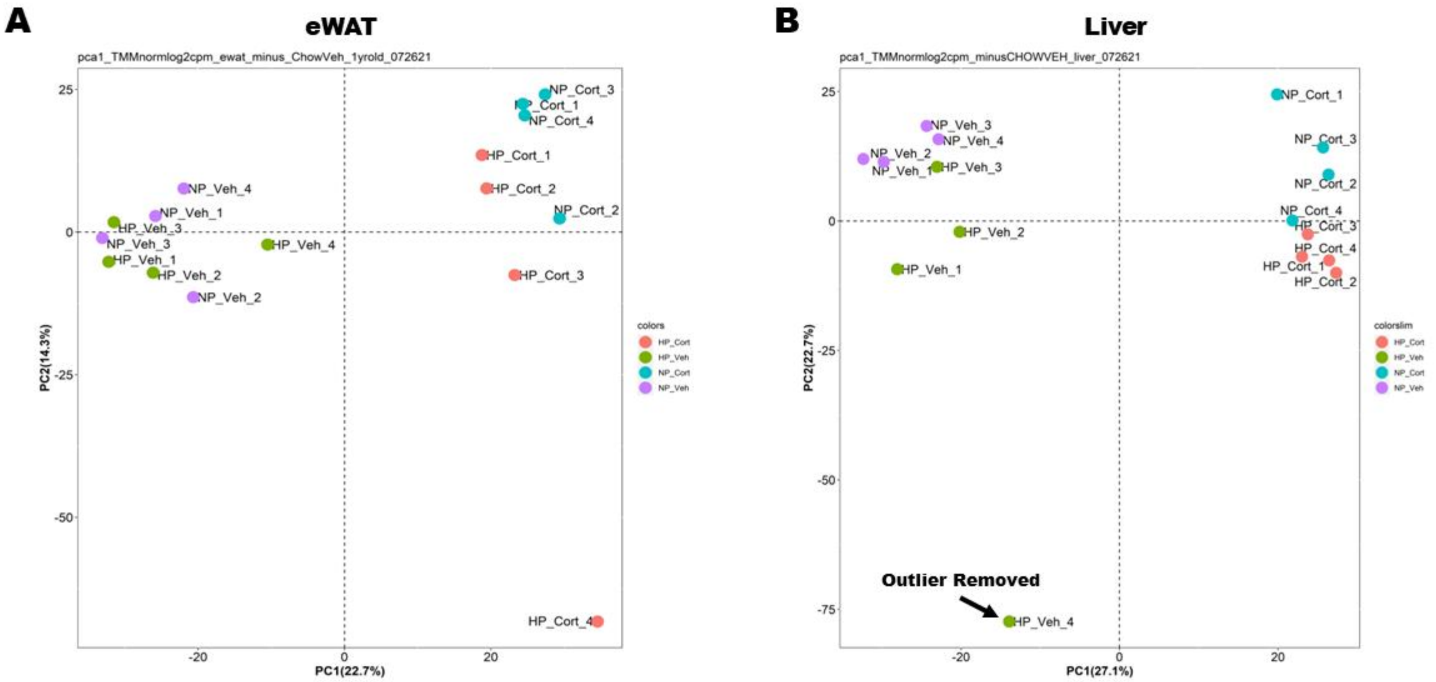

Supplemental Figure S1. Principle components analysis of RNAseq data from epididymal white adipose tissue (eWAT; panel A) and liver (panel B) from mice that were given corticosterone (Cort) or 1% ethanol vehicle (Veh) control in the drinking water. All mice were fed either a diet containing normal protein (NP) or high protein (HP). n=4/group.

SUPPLEMENTAL TABLE S1

Parameter	Normal Protein Diet	High Protein Diet
Protein	19 grams (20% kcal)	58 grams (60% kcal)
Carbohydrate	67 grams (70% kcal)	29 grams (30% kcal)
Fat	4 grams (10% kcal)	4 grams (10% kcal)
**Ingredients**		
Whey Protein Isolate	203 grams (812 kcal)	609 grams (2436 kcal)
Corn Starch	505 grams (2020 kcal)	99 grams (396 kcal)
Maltodextrin 10	45 grams (180 kcal)	45 grams (180 kcal)
Sucrose	150 grams (600 kcal)	150 grams (600 kcal)
Cellulose (BW 200)	50 grams (0 kcal)	50 grams (0 kcal)
Soybean Oil	25 grams (225 kcal)	25 grams (225 kcal)
Lard	20 grams (180 kcal)	20 grams (180 kcal)
Vitamin Mix V10001	10 grams (40 kcal)	10 grams (40 kcal)
**Total**	1055 grams (4057 kcal)	1055 grams (4057 kcal)

Supplementary Table S1. Dietary formulations used in this study. Note that corn starch was reduced in the high protein diet relative to the normal protein diet to keep the formulations isocaloric. Sucrose and maltodextrin 10 were matched between the diets.
